# Frequency and Spectrum of Ocular Manifestations Among Patients With Systemic Autoimmune Rheumatic Diseases: A Retrospective Cross-Sectional Study

**DOI:** 10.7759/cureus.116972

**Published:** 2026-09-26

**Authors:** Muhammad Mansoor Ali, Masem Afzal, Muhammad Hassan Saeed, Ahmad Hassan

**Affiliations:** 1 Medicine, Al-Aleem Medical College, Gulab Devi Teaching Hospital, Lahore, PAK; 2 Acute Medicine, Wrexham Maelor Hospital, Wrexham, GBR; 3 Rheumatology, Al-Aleem Medical College, Gulab Devi Teaching Hospital, Lahore, PAK; 4 Emergency Medicine, Shaikh Zayed Hospital, Lahore, PAK

**Keywords:** adult internal medicine, dry eye disease, internal medicine, ocular involvement, ocular manifestations, rheumatology, scleritis, systemic autoimmune rheumatic diseases, uveitis

## Abstract

Introduction: Systemic autoimmune rheumatic diseases may cause a wide range of ocular manifestations, ranging from dry eye disease to potentially vision-threatening inflammatory and vascular complications. Data regarding the frequency and spectrum of ocular involvement among patients with systemic autoimmune rheumatic diseases in Pakistan remain limited. This study aimed to determine the frequency and spectrum of documented ocular manifestations among patients with systemic autoimmune rheumatic diseases.

Methods: A retrospective, hospital-based cross-sectional study was conducted at Gulab Devi Teaching Hospital, Lahore, Pakistan, using medical records from May 2026 to August 2026. Adult patients aged ≥18 years with an established systemic autoimmune rheumatic disease who attended rheumatology or ophthalmology outpatient departments or were admitted to any inpatient department were eligible. Patients with insufficient records to determine the underlying rheumatic diagnosis or ocular status were excluded. Each patient was counted only once. Data included demographics, rheumatic diagnosis, disease duration where documented, and presence and type of ocular manifestation. Ocular involvement was defined as at least one documented ocular manifestation. Data were analyzed using SPSS Statistics (IBM Corp., Armonk, NY, USA). Categorical variables were summarized as frequencies and percentages.

Results: A total of 210 unique eligible patients were included. The mean age was 37.6 ± 12.8 years, and 164 (78.1%) were female. Rheumatoid arthritis was the most common rheumatic disease (82, 39.0%), followed by systemic lupus erythematosus (SLE) (57, 27.1%) and spondyloarthritis (26, 12.4%). Overall, 94 (44.8%) patients had at least one documented ocular manifestation. Dry eye/keratoconjunctivitis sicca was the most frequently documented manifestation (39, 18.6%), followed by uveitis (24, 11.4%), scleritis (13, 6.2%), episcleritis (7, 3.3%), cataract (6, 2.9%), peripheral ulcerative keratitis (5, 2.4%), glaucoma (5, 2.4%), and retinal vasculitis (4, 1.9%).

Conclusion: Ocular manifestations were frequently documented among patients with systemic autoimmune rheumatic diseases, with a broad spectrum of ocular involvement observed across different rheumatological diagnoses. The findings highlight the importance of recognizing ocular disease in these patients and support collaboration between rheumatology and ophthalmology services. Further prospective multicenter studies using standardized ophthalmological assessment are warranted.

## Introduction

Systemic autoimmune rheumatic diseases are chronic inflammatory disorders that can affect multiple organ systems, including the eye. Ocular involvement may contribute substantially to morbidity and impaired quality of life in affected patients [1]. The spectrum of ocular manifestations varies according to the underlying rheumatic disease and may involve the ocular surface, anterior segment, posterior segment, or optic nerve [1]. Commonly described manifestations include dry eye disease, keratoconjunctivitis sicca, uveitis, episcleritis, scleritis, keratitis, peripheral ulcerative keratitis, retinal vascular abnormalities, and other inflammatory or structural ocular complications [2]. Uveitis is particularly associated with spondyloarthritis and may occur early in the course of disease, sometimes preceding other systemic manifestations [3]. Rheumatoid arthritis has been associated with several ocular manifestations, particularly dry eye disease, episcleritis, scleritis, peripheral ulcerative keratitis, and corneal ulceration [4]. In Sjögren's syndrome, autoimmune involvement of the lacrimal glands contributes to aqueous-deficient dry eye disease, which represents one of the characteristic ocular manifestations of the condition [5].

Systemic lupus erythematosus (SLE) can involve both the ocular surface and posterior segment, with manifestations including dry eye, retinal vascular abnormalities, retinopathy, and choroidal involvement [6]. Recent studies have also demonstrated that some ocular abnormalities in SLE may be clinically subtle or detectable only with advanced ophthalmic imaging, highlighting the potential importance of ocular assessment even when overt ocular symptoms are absent [7]. Ocular involvement is also clinically important in Behçet disease, in which uveitis and retinal vascular inflammation can result in significant visual morbidity [8]. Similarly, patients with ANCA-associated vasculitis may develop a range of ocular manifestations, including inflammatory and retinal complications [9]. Systemic sclerosis can involve both the anterior and posterior segments of the eye, with reported manifestations including dry eye, conjunctival abnormalities, retinal microvascular changes, and optic nerve abnormalities [10]. Because ocular disease may precede or accompany systemic inflammatory disease, recognition of ocular manifestations can facilitate earlier diagnosis, appropriate referral, and multidisciplinary management [2].

Despite the recognized association between systemic autoimmune rheumatic diseases and ocular involvement, the frequency and spectrum of ocular manifestations vary substantially across rheumatic diseases and study populations [11]. In addition, available studies have often focused on individual rheumatic diseases or specific ocular manifestations, with fewer studies describing the overall ocular spectrum across a broad rheumatology population [11]. Data describing ocular manifestations among patients with systemic autoimmune rheumatic diseases in Pakistan remain limited. Therefore, this study aimed to determine the frequency and spectrum of documented ocular manifestations among patients with systemic autoimmune rheumatic diseases and to describe their distribution according to the underlying rheumatic disease.

## Materials and methods

Study design and setting

We conducted a retrospective, hospital-based cross-sectional study using medical records of patients with systemic autoimmune rheumatic diseases to determine the frequency and spectrum of documented ocular manifestations. The study was conducted at Gulab Devi Teaching Hospital, Lahore, Pakistan. Medical records of eligible patients encountered between May 2026 and August 2026 were reviewed. Adult patients with established systemic autoimmune rheumatic diseases who attended the rheumatology or ophthalmology outpatient departments or were admitted to any inpatient department of the hospital during the study period were eligible for inclusion. A total of 210 eligible patients were included in the study based on the available eligible records during the study period. Patients with multiple clinical encounters or records across different departments were counted only once.

Inclusion and exclusion criteria 

Adult patients aged 18 years or older with an established diagnosis of a systemic autoimmune rheumatic disease documented in the medical record were eligible for inclusion. The study population was not restricted to specific individual rheumatic diagnoses; all patients meeting the general eligibility criterion were considered for inclusion. The presence or absence of an ocular manifestation was not an inclusion criterion, and patients were included regardless of their ocular status. Underlying rheumatic disease diagnoses were based on the established diagnoses documented in the patients' medical records by the treating physician. Classification criteria were not retrospectively reapplied for the purposes of this study.

Patients younger than 18 years, those without an established systemic autoimmune rheumatic disease, or those with insufficient medical records to determine the underlying rheumatic diagnosis were excluded. Each patient was included only once, regardless of whether the patient had encounters in more than one clinical setting during the study period. Comorbid medical conditions were not used as exclusion criteria.

Study outcomes

The primary outcome was the frequency of documented ocular manifestations among patients with systemic autoimmune rheumatic diseases. Secondary outcomes included the spectrum of ocular manifestations and the distribution of ocular manifestations according to the underlying rheumatic disease. For the primary outcome, ocular involvement was defined as the presence of at least one documented ocular condition or manifestation recorded in the patient's medical record. Documented manifestations included dry eye/keratoconjunctivitis sicca, uveitis, scleritis, episcleritis, peripheral ulcerative keratitis, retinal vasculitis, cataract, glaucoma, and other physician-documented ocular conditions. Patients without a documented ocular manifestation were classified as having no documented ocular involvement.

Data collection

A structured data collection proforma (see Appendices) was developed for the study and used to systematically collect relevant demographic, rheumatological, and ophthalmological information. Data were obtained from the medical records of eligible patients attending the rheumatology and ophthalmology outpatient departments, as well as from patients with systemic autoimmune rheumatic diseases admitted to any inpatient department of the hospital during the study period. After eligible patients were identified based on their age and underlying systemic autoimmune rheumatic disease, their medical records were reviewed for the presence or absence of documented ocular manifestations and, when present, the specific type of ocular manifestation. The proforma included age, sex, underlying systemic autoimmune rheumatic disease, disease duration where documented, presence or absence of a documented ocular manifestation, and type of ocular manifestation.

Ocular manifestations were identified from documented ophthalmology diagnoses and relevant clinical records. The recorded ocular conditions included dry eye/keratoconjunctivitis sicca, uveitis, scleritis, episcleritis, keratitis, peripheral ulcerative keratitis, retinal vasculitis, optic neuritis, cataract, glaucoma, and other documented ocular conditions. Dry eye/keratoconjunctivitis sicca was recorded when documented as a clinical diagnosis or finding in the available medical record. Objective dry eye testing, such as Schirmer testing or tear-film assessment, was not performed specifically for this retrospective study.

No examinations or investigations were performed specifically for the purpose of this study. Dry eye/keratoconjunctivitis sicca was recorded when documented as a clinical diagnosis or finding in the available medical record. Objective dry-eye testing was not performed specifically for this retrospective study.

Statistical analysis

Data were entered and analyzed using SPSS Statistics version 26.0 (IBM Corp., Armonk, NY, USA). Continuous variables were summarized using mean and standard deviation, while categorical variables were summarized as frequencies and percentages. The frequency of ocular involvement was calculated as the proportion of patients with at least one documented ocular manifestation among the total study population. The frequencies and percentages of individual ocular manifestations were also calculated for ocular involvement within each rheumatic disease category. The analysis was descriptive, and no inferential statistical tests were performed. Because individual patients could have more than one ocular manifestation, individual ocular diagnoses were not considered mutually exclusive, and their frequencies were not expected to sum up to the total number of patients with ocular involvement.

Ethical considerations

The study was conducted in accordance with the ethical principles for research involving human participants. Ethical approval was obtained from the Institutional Review Board of Al-Aleem Medical College before commencement of the study (approval no. AAMC/IRB/EA/17/2026). Informed consent was obtained, patient confidentiality was maintained throughout the study, and identifying information was not included in the study database or analysis.

## Results

Patient characteristics

A total of 210 patients with established systemic autoimmune rheumatic diseases were included in the study. The mean age of the study population was 37.6 ± 12.8 years, and 164 (78.1%) patients were female. Rheumatoid arthritis was the most common underlying rheumatic disease, followed by SLE and spondyloarthritis. The distribution of underlying rheumatic diseases is shown in Table 1.

**Table 1:** Demographic characteristics and distribution of underlying rheumatic diseases SLE: Systemic lupus erythematosus

Variable		N (%), (total n=210)
Demographic characteristics	Age, mean ± SD	37.6 ± 12.8
	Female	164 (78.1)
	Male	46 (21.9)
Underlying rheumatic disease	Rheumatoid arthritis	82 (39.0)
	SLE	57 (27.1)
	Spondyloarthritis	26 (12.4)
	Systemic sclerosis	13 (6.2)
	Sjögren's syndrome	10 (4.8)
	Systemic vasculitis	7 (3.3)
	Behçet's disease	5 (2.4)
	Mixed connective tissue disease	4 (1.9)
	Idiopathic inflammatory myopathies	3 (1.4)
	Adult-onset Still's disease	3 (1.4)

Frequency of ocular manifestations

Among the 210 patients, 94 (44.8%) had at least one documented ocular manifestation, while 116 (55.2%) had no documented ocular involvement. Dry eye/keratoconjunctivitis sicca was the most frequently documented ocular manifestation, occurring in 39 (18.6%) patients, followed by uveitis in 24 (11.4%), scleritis in 13 (6.2%), episcleritis in seven (3.3%), cataract in six (2.9%), peripheral ulcerative keratitis in five (2.4%), glaucoma in five (2.4%), and retinal vasculitis in four (1.9%) patients (Table 2). The frequency of individual ocular manifestations does not sum up to the total number of patients with ocular involvement because some patients had more than one documented ocular manifestation. Thus, the 94 patients represent the number of individuals with at least one ocular manifestation, whereas the individual rows in Table 2 represent the frequency of each specific manifestation. Accordingly, the percentages for individual manifestations were calculated using the total study population of 210 patients and are not mutually exclusive; therefore, their percentages are not expected to sum up to 100%.

**Table 2:** Frequency of documented ocular manifestations among study participants *The denominator for percentages is the total study population (N=210). “Any ocular manifestation” represents the number of patients with at least one documented ocular manifestation. Individual ocular manifestations were not mutually exclusive, as some patients had more than one manifestation; therefore, the frequencies and percentages of individual manifestations do not sum up to the total number of patients with ocular involvement or to 100%.

Ocular manifestation*	N (%)
Any ocular manifestation	94 (44.8)
Dry eye/keratoconjunctivitis sicca	39 (18.6)
Uveitis	24 (11.4)
Scleritis	13 (6.2)
Episcleritis	7 (3.3)
Cataract	6 (2.9)
Peripheral ulcerative keratitis	5 (2.4)
Glaucoma	5 (2.4)
Retinal vasculitis	4 (1.9)

Ocular manifestations according to rheumatic disease

Among patients with rheumatoid arthritis, 37 of 82 (45.1%) had at least one documented ocular manifestation. Among patients with SLE, 27 of 57 (47.4%) had ocular involvement, while ocular manifestations were documented in 14 of 26 (53.8%) patients with spondyloarthritis. Ocular involvement was documented in six of 13 (46.2%) patients with systemic sclerosis, five of 10 (50.0%) patients with Sjögren's syndrome, three of seven (42.9%) patients with systemic vasculitis, two of five (40.0%) patients with Behçet's disease, and zero of 10 (0.0%) patients with other autoimmune rheumatic diseases, including mixed connective tissue disease (n=4), idiopathic inflammatory myopathies (n=3), and adult-onset Still's disease (n=3). The distribution of ocular involvement according to the underlying rheumatic disease is presented in Table 3. Overall, ocular manifestations were documented across multiple systemic autoimmune rheumatic diseases, with dry eye/keratoconjunctivitis sicca being the most frequent manifestation, followed by uveitis and scleritis. These findings describe observed frequencies and associations and do not establish a causal relationship between systemic autoimmune rheumatic diseases and ocular manifestations.

**Table 3:** Frequency of ocular involvement according to underlying rheumatic disease *Other autoimmune rheumatic diseases included mixed connective tissue disease (n=4), idiopathic inflammatory myopathies (n=3), and adult-onset Still's disease (n=3). Ocular involvement was defined as the presence of at least one documented ocular manifestation. Percentages represent the proportion of patients within each rheumatic disease category. SLE: Systemic lupus erythematosus

Rheumatic disease	N	Ocular involvement, n (%)
Rheumatoid arthritis	82	37 (45.1)
SLE	57	27 (47.4)
Spondyloarthritis	26	14 (53.8)
Systemic sclerosis	13	6 (46.2)
Sjögren's syndrome	10	5 (50.0)
Systemic vasculitis	7	3 (42.9)
Behçet's disease	5	2 (40.0)
Other autoimmune rheumatic diseases*	10	0 (0.0)
Total	210	94 (44.8)

## Discussion

In this retrospective cross-sectional study of 210 patients with systemic autoimmune rheumatic diseases at a tertiary-care hospital in Lahore, Pakistan, ocular manifestations were assessed across a range of rheumatological conditions. The study describes a broad spectrum of documented ocular manifestations within this hospital-based population and highlights the importance of recognizing ocular disease as part of systemic rheumatological care.

The overall frequency of documented ocular involvement in our study was 44.8%. This descriptive estimate is broadly consistent with previous literature indicating that ocular manifestations are reported among patients with rheumatic diseases, although reported frequencies vary according to the underlying disease, study population, method of ascertainment, and definition of ocular involvement [1,11]. In a retrospective cohort of 1,529 rheumatology outpatients, Altenberger et al. reported an ophthalmologic diagnosis in 26.9% of patients, illustrating the variability in reported frequency across different clinical populations and methods of case ascertainment [11]. Their study involved patients identified through both rheumatology and ophthalmology services, whereas our study focused specifically on documented ocular manifestations among patients with established systemic autoimmune rheumatic diseases.

The relatively high frequency observed in our cohort may partly reflect the hospital-based nature of the study and the inclusion of patients identified through both rheumatology and ophthalmology services. In the study by Altenberger et al., inflammatory non-infectious ophthalmologic disease was documented in 24.2% of patients with rheumatoid arthritis and 29.3% of those with peripheral spondyloarthritis, further demonstrating that the observed frequency of ocular disease depends on the characteristics of the population studied [11].

Dry eye/keratoconjunctivitis sicca was the most frequently documented ocular manifestation in our cohort. This finding is consistent with the established association between systemic autoimmune disease and ocular surface disease, particularly in Sjögren's syndrome [5]. Sjögren's syndrome is characterized by autoimmune exocrine gland dysfunction, including lacrimal gland involvement, resulting in aqueous-deficient dry eye disease [5]. The prominence of dry eye in our cohort is therefore biologically plausible, particularly given the inclusion of patients with Sjögren's syndrome as well as other systemic autoimmune rheumatic diseases.

Rheumatoid arthritis is also associated with dry eye and other ocular surface abnormalities, and ocular manifestations may occur alongside more destructive inflammatory conditions such as scleritis and peripheral ulcerative keratitis [4]. In a study of 196 patients with rheumatoid arthritis, ocular manifestations were documented in 39%, with dry eye being the most common manifestation, occurring in 28%; episcleritis, scleritis, and peripheral keratitis were reported in 3%, 2%, and 1% of patients, respectively [12]. These findings are broadly consistent with the spectrum observed in our cohort, although direct comparison should be made cautiously because the cited study involved patients undergoing detailed ophthalmic examination, whereas our study relied on documented clinical records.

Uveitis represented another important manifestation in our cohort. This finding is clinically relevant because uveitis is strongly associated with several systemic inflammatory rheumatic diseases, particularly spondyloarthritis [3]. A recent review reported that uveitis occurs in approximately 22% to 24.5% of patients with spondyloarthritis and that acute anterior uveitis represents approximately 90% of uveitis in this population [13]. In the cohort reported by Altenberger et al., anterior uveitis was documented in 16.5% of patients with spondyloarthritis compared with none of the patients with rheumatoid arthritis [11].

Spondyloarthritis-associated uveitis is typically acute anterior uveitis and may recur over time, with recurrent disease carrying a risk of ocular complications and visual impairment [13]. The review by Jacquot et al. noted that in ankylosing spondylitis, acute uveitis was reported to be unilateral in approximately 85% of cases and acute in 93% of cases [13]. The presence of uveitis in patients with rheumatic disease is particularly important because ocular inflammation can occasionally precede recognition of the underlying systemic disease [3,13].

Our findings also demonstrate ocular involvement among patients with SLE. Previous literature has described a broad range of ocular manifestations in SLE, including dry eye, retinopathy, choroidopathy, retinal vascular abnormalities, and other posterior segment changes [6]. Retinopathy has been reported in approximately 12% of patients with SLE, although reported frequencies vary with disease activity, treatment, and the methods used to detect ocular abnormalities [6].

Recent studies have further shown that ocular involvement in SLE may be clinically subtle. The literature reviewed by Meng et al. indicates that ocular involvement may occur in approximately one-third of patients with SLE and that multimodal imaging can identify subclinical retinal and choroidal abnormalities even in patients without overt ocular manifestations [7]. These findings suggest that conventional clinical assessment may not capture the entire spectrum of ocular involvement. The presence of retinal manifestations in SLE is also clinically important because ocular fundus abnormalities may correlate with systemic disease activity and vascular involvement [7]. However, because our study was based on documented clinical records and did not include systematic multimodal retinal imaging, subclinical ocular abnormalities may have been underrecognized.

In patients with Behçet's disease, ocular involvement is particularly important because uveitis may be associated with retinal ischemia, vascular occlusion, macular complications, and progressive visual impairment [8,14]. In a multicenter study of 175 patients with ocular Behçet's disease, retinal vascular occlusions were identified in 30.9% of patients, involving 80 eyes, demonstrating the potential severity of posterior segment vascular disease in this condition [8].

Similarly, ocular manifestations in ANCA-associated vasculitis encompass a diverse range of inflammatory and vascular abnormalities [9]. In a retrospective multicenter study of 533 patients with ANCA-associated vasculitis, ocular complications were observed in 20.64% of patients, with reported frequencies of 36.67% in granulomatosis with polyangiitis, 7.37% in microscopic polyangiitis, and 18.18% in eosinophilic granulomatosis with polyangiitis [9]. These findings illustrate the substantial variation in ocular involvement across different vasculitic disorders.

Systemic sclerosis patients may also develop ocular abnormalities involving both anterior and posterior ocular structures. Recent reviews have described dry eye, conjunctival and eyelid abnormalities, retinal microvascular changes, and optic nerve abnormalities in affected patients [10,15]. Carlà et al. reported that eyelid abnormalities may affect up to two-thirds of patients with systemic sclerosis, while earlier studies using retinal imaging reported vascular abnormalities in approximately half of affected patients [10].

In addition to systemic sclerosis, ocular surface disease is an important manifestation across autoimmune rheumatic disorders. In Sjögren's syndrome, dry eye disease results primarily from autoimmune-mediated lacrimal gland dysfunction and may require comprehensive ophthalmological assessment for accurate diagnosis and characterization [16]. This highlights the importance of recognizing ocular surface abnormalities as part of the broader spectrum of ocular involvement in systemic autoimmune rheumatic diseases.

An important strength of this study is the inclusion of multiple systemic autoimmune rheumatic diseases within the same study population. This allowed a description of the spectrum of documented ocular manifestations across different rheumatological conditions within a hospital-based population. The study also provides descriptive data from a Pakistani hospital population, where published information on the frequency and spectrum of ocular manifestations across multiple systemic autoimmune rheumatic diseases remains limited.

Despite the limitations inherent to its retrospective, single-center design, this study provides useful local data regarding the frequency and spectrum of documented ocular manifestations among patients with systemic autoimmune rheumatic diseases. Because the study relied on existing medical records and did not perform standardized ophthalmic examinations or additional investigations specifically for research purposes, the observed frequency may not represent the true prevalence of all ocular abnormalities, particularly asymptomatic or subclinical disease. The findings support close collaboration between rheumatologists and ophthalmologists, particularly for patients with diseases known to carry a higher risk of ocular inflammation or vision-threatening ocular disease [1,3].

Limitations

This study has several limitations that should be considered when interpreting the findings. The retrospective design means that the study relied on the quality and completeness of existing medical records, and ocular manifestations that were not documented may therefore have been missed. Ocular involvement was based on documented clinical diagnoses rather than standardized ophthalmological examination of every participant, which may have resulted in underestimation of asymptomatic or subclinical ocular abnormalities. The single-center setting in Lahore may also limit the generalizability of the findings to other populations and healthcare settings in Pakistan. In addition, given the cross-sectional nature of the study, the observed associations should not be interpreted as causal relationships, and temporal relationships between the underlying rheumatic diseases and ocular manifestations could not be established. Variations in documentation practices and the extent of ophthalmological assessment may have also influenced the observed frequency and spectrum of ocular manifestations. The study was conducted at a single teaching hospital in Lahore city; therefore, the findings should be interpreted within this specific geographical and healthcare setting and may not be directly generalizable to patients with systemic autoimmune rheumatic diseases across Pakistan.

## Conclusions

Ocular manifestations were frequently documented among patients with systemic autoimmune rheumatic diseases in this hospital-based cohort, with a broad spectrum of ocular involvement observed across different rheumatological diagnoses. Dry eye/keratoconjunctivitis sicca and uveitis were among the commonly identified manifestations. These findings highlight the importance of awareness of ocular involvement in patients with systemic autoimmune rheumatic diseases and support effective collaboration between rheumatology and ophthalmology services. Further future multicenter prospective studies incorporating standardized ophthalmological assessments are warranted to better characterize the burden of both clinically apparent and subclinical ocular involvement and to establish consistent approaches to screening and characterization of ocular manifestations across systemic autoimmune rheumatic diseases.

## Figures and Tables

**Figure 1:**
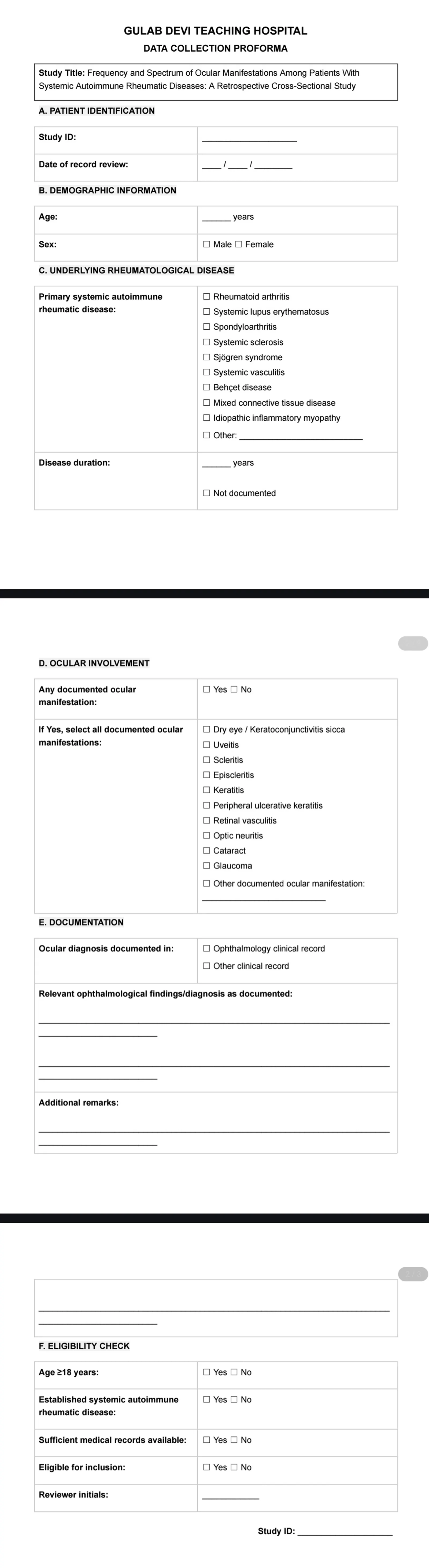
Data collection proforma
